# A Novel Point Cloud Encoding Method Based on Local Information for 3D Classification and Segmentation

**DOI:** 10.3390/s20092501

**Published:** 2020-04-28

**Authors:** Yanan Song, Liang Gao, Xinyu Li, Weiming Shen

**Affiliations:** State Key Lab. of Digital Manufacturing Equipment & Technology, Huazhong University of Science and Technology, Wuhan 430074, China; yanansongwell@163.com (Y.S.); gaoliang@mail.hust.edu.cn (L.G.); lixinyu@mail.hust.edu.cn (X.L.)

**Keywords:** Internet of Things, point cloud, deep learning, 3D classification, segmentation

## Abstract

Deep learning is robust to the perturbation of a point cloud, which is an important data form in the Internet of Things. However, it cannot effectively capture the local information of the point cloud and recognize the fine-grained features of an object. Different levels of features in the deep learning network are integrated to obtain local information, but this strategy increases network complexity. This paper proposes an effective point cloud encoding method that facilitates the deep learning network to utilize the local information. An axis-aligned cube is used to search for a local region that represents the local information. All of the points in the local region are available to construct the feature representation of each point. These feature representations are then input to a deep learning network. Two well-known datasets, ModelNet40 shape classification benchmark and Stanford 3D Indoor Semantics Dataset, are used to test the performance of the proposed method. Compared with other methods with complicated structures, the proposed method with only a simple deep learning network, can achieve a higher accuracy in 3D object classification and semantic segmentation.

## 1. Introduction

Big data from different sensors are the basis for the Internet of Things (IoT) to play its own advantages. Point cloud is an important 3D data format that is widely used in 3D semantic segmentation [1,2] and 3D object detection [3,4]. Moreover, point cloud is a raw output format of many 3D capturing devices such as depth cameras and LiDAR sensors. They are all important components in IoT-based smart systems. However, point cloud is usually unordered and unstructured, which makes it difficult to use these points effectively. Deep learning has been successfully used to process multiple data such as fault signals [5,6] and images [7,8]. However, it is not easy to use deep learning to directly process the point cloud because of its irregular format.

To utilize the deep learning method, some researchers converted raw point cloud data to regular formats such as 3D voxel grids [9] and multi-view renderings [10]. However, this conversion not only increases the network complexity, but also loses some useful information of the data. PointNet [11] was a pioneer that used the point cloud as the direct input of the deep learning network. PointNet first extracts features of each point independently, and then, the extracted features are integrated together to obtain the global information of the point cloud. Because PointNet extracts the features separately from individual points, it does not capture the local information of the point cloud.

Generally speaking, context awareness is conducive to improving the performance of the algorithm [12]. Utilizing local information has an important impact on the success of the deep learning method that often chooses convolutional neural networks (CNN) as the framework. The local information of the data facilitates the deep learning network to identify fine-grained features. The lower level of the deep learning network generally provides a smaller receptive field, while the higher level provides a larger receptive field. This gives the deep learning network an ability to capture the low level location information and the high level semantic information. Therefore, it is very important to utilize the local information when designing an algorithm based on the deep learning network.

To address this requirement, PointNet++ [13] introduced the local information of the point cloud into the deep learning network. The method partitioned the point cloud into overlapping local regions. The local features were extracted by applying PointNet recursively to these local regions. Landrieu and Simonovsky [14] designed a superpoint graph structure to effectively represent the relationship between different object parts. Li et al. [15] proposed a SO-Net structure to utilize the spatial distribution of the point cloud. Although these methods can make use of the local information, the models become very complicated compared with PointNet.

This paper proposes an effective method to encode each point into a feature representation with local information. This method facilitates a deep learning network to utilize the local information. An axis-aligned cube is first applied to the point cloud to search for a local spatial region that represents the local information. Second, some points are randomly selected from these points in the local region to construct the feature representation of a point. Each point is then encoded into a two-dimensional vector by randomly arranging those selected points. A simple deep learning network is finally designed to directly process the feature representation of all points. The well-known datasets ModelNet40 [16] shape classification benchmark and Stanford 3D Indoor Semantics Dataset (S3DIS) [17] are respectively used to test the proposed method for 3D object classification and semantic segmentation. Experimental results show that the proposed method achieves high accuracies on these two tasks even if only a simple deep learning network is used.

In the remainder of this paper, previous studies are summarized in Section 2. Details about the proposed method are introduced in Section 3. Experiments based on ModelNet40 and S3DIS are provided in Section 4. Conclusions and future work are discussed in Section 5.

## 2. Related Work

The methods for processing 3D data can be divided into two categories based on the feature representation. The traditional methods rely on hand-designed features. Deep learning is a new method to learn the feature representation directly from raw data.

Most traditional methods for 3D data are designed according to the handcrafted features. The Light Field descriptor (LFD) [18] extracted from 4D light fields was the representation of 3D objects, and it was used to measure the similarity between 3D models. The Spherical Harmonic descriptor (SPH) [19] was used to improve the matching performance of orientation dependent descriptors. The fast point feature histogram was used to retrieve three-dimensional objects by using fisher encodings [20]. For most application scenarios, the feature representation of the point cloud was often encoded by using statistical properties [21]. However, the optimal feature representation is often different on specific tasks, and it is not easy to choose the optimal feature for a specific task.

Recently, the end-to-end learning algorithms have been proposed for 3D data analysis. The early approaches that processed 3D data using deep learning were found in the literature [22,23], which handled 3D shape recognition task. The 3D ShapeNets [16] learned a probability distribution of binary variables of 3D shapes from multiple categories and arbitrary poses. Qi et al. [24] used an auxiliary task that predicted object classes from partial sub-volumes to improve the performance of the volumetric CNN. In these methods, 3D CNN is the main network frame that requires the voxel grids. These voxels are obtained by transforming the format of the raw point cloud. Unfortunately, these methods consume a large amount of computer memory. The size of the volumetric representation and the depth of the CNN are limited.

Researchers have proposed various methods to address this problem. Li et al. [25] proposed a field probing filter to extract features from volumetric fields. Riegler et al. [26] proposed an OctNet to hierarchically partition the 3D input space using a set of unbalanced octrees. The computational resources were dynamically focused based on the input 3D structure. Engelcke et al. [27] designed a sparse convolutional layer based on a voting strategy. Their method can efficiently process full 3D point clouds. However, the advantages of these methods are reduced when the point clouds are very large.

Other research efforts to ease the computational intensity are to render the 3D data into 2D images. The 3D point clouds were projected into 2D images by Pang and Neumann [28], and the point cloud detection was completed in a 2D space using CNN. A cylinder projection around its principle axis was operated by Shi et al. [29] for each 3D shape. A 2D CNN was designed by Su et al. [30] to recognize the rendered views, and the information from different views was integrated to form a single compact shape descriptor. Although these methods using multi-view CNN require less computational memory, some information is lost in the process of converting the point cloud into 2D images. Moreover, these methods perform poorly in some tasks such as scene understanding.

To avoid complex network structures and data rendering, the methods that directly process unordered point clouds are also proposed. PointNet [11] independently extracts the features of each point, and all of the features from the point cloud are then aggregated into a global feature by max pooling. PointNet is highly efficient and effective for many 3D data analysis tasks such as classification and segmentation. However, this method focuses on a single point and ignores the use of local geometric contexts of points. The improved PointNet was called as PointNet++, which was used to solve this problem [13]. PointNet++ was a hierarchical neural network that processed the point cloud in a hierarchical fashion. It first extracted the local features from the small neighborhoods, and these features were then grouped into larger units. Although local geometric contexts are considered in PointNet++, it requires extra computations. Other methods that directly process point clouds are studied based on PointNet. Huang et al. [31] projected the features of unordered points into an ordered sequence of feature vectors by using a slice pooling layer, and the traditional recurrent neural networks were then applied to these features. Wang et al. [32] proposed a similarity group proposal network to directly learn 3D instance-aware semantic segmentation from the point cloud. Although these methods can directly handle point clouds, they require novel models that are complicated compared with the PointNet.

Different from the methods mentioned above, the proposed method only uses a simple network whose complexity is lower than that of the PointNet. The proposed method processes the raw point cloud independently of the network structure. It can handle large-scale point clouds effectively and adapt to most point cloud data analysis tasks. Moreover, the local information of a point cloud is captured by utilizing the neighborhood points, which facilitates the network to recognize the fine-grained features of the objects.

## 3. Proposed Method for 3D Classification and Segmentation

A point cloud is a set of points, and each point interacts with its surrounding points. It is reasonable to consider the spatial location information and the influence of the surrounding points when constructing the feature representation of a point. In addition, the deep learning network requires a regular input format, and it is necessary to keep the feature dimensions of each point the same.

Taking into account the above factors, a point cloud encoding method is proposed to build the feature representation with the local information. The local information is represented using a local region that is an axis-aligned cube. The feature representation of each point is a two-dimensional vector. The vector is obtained using some points that are randomly chosen from the local region.

**Figure 1:**
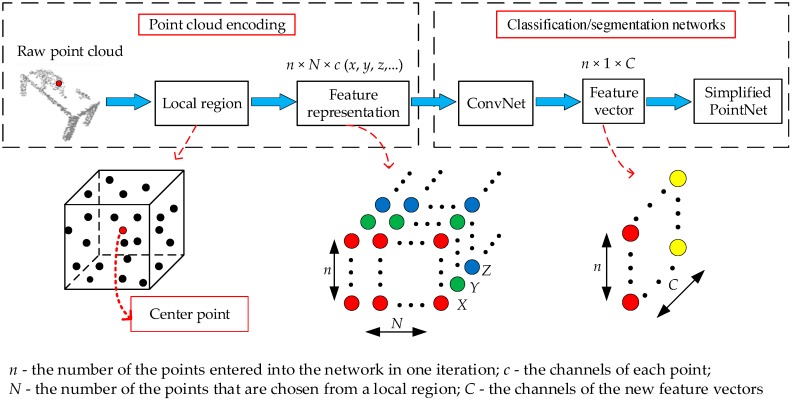
The overall framework of the proposed method.

PointNet is a pioneer in directly processing point clouds. To verify the effectiveness of the proposed point cloud encoding strategy, only a simple deep learning network is designed on the basis of PointNet. The network is used to process the feature representation of each point. The overall framework of the proposed method is shown in Figure 1, which consists of a point cloud encoding block and a classification/segmentation networks block. Details of the proposed method are provided separately in the following sub-sections.

Different from the PointNet, the input transformation, feature transformation, and regularization loss are removed in the proposed deep learning architectures. The complexity of the proposed structure is much lower than PointNet. In addition, the data input to the network is the encoded feature representation, not the point coordinates used by PointNet. The feature representation contains abundant local information, which is helpful to improve the recognition accuracy.

### 3.1. Search for Local Region

Searching for a local region is actually a process that indexes spatial points from the point cloud. A cube region is often used in the Octree method [33]. The method partitions the point cloud by recursively subdividing it into eight regions, which improves the speed of indexing 3D data. The cube is also used to search for local regions of the point cloud in the proposed method.

**Figure 2:**
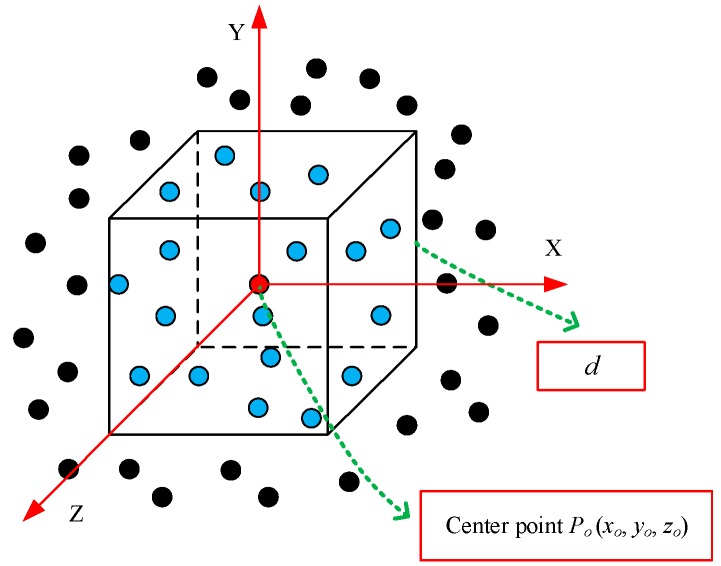
The local region searched by an axis-aligned cube.

It is assumed that a point cloud is represented as a set *G* = {*P_i_*| *i* =1, 2, …, *n*}, *P_i_* is represented using (*x_i_*, *y_i_*, *z_i_*) coordinates and other extra channels such as color and normalized location. The process of searching for a local region is shown in Figure 2. The local region of a point *P_o_* (*x_o_*, *y_o_*, *z_o_*) is searched using a cube with side length *d*. The point *P_o_* is located at the center of the cube and is called the center point. The range of the local region for *P_o_* is determined based on (1). The cube is aligned with the coordinate axis. The axis-aligned cube helps determine whether a point is in the local region. If the three coordinate values of a point satisfy (1) at the same time, then the point is considered to belong to the local region.

(1) $$\left\{ \begin{array}{l} \left[x_{o}-d/2,\;x_{o}+d/2\right]\\ \left[y_{o}-d/2,\;y_{o}+d/2\right]\\ \left[z_{o}-d/2,\;z_{o}+d/2\right] \end{array}\right.$$

The local region is only used to search for the points around an encoded point, and it provides the local information for the encoding features. The coordinate values are used to determine whether a point is in the local region. It is unnecessary to calculate the distances between points during the process of searching for the local region. Compared with other methods that depend on the distance information [34], the proposed method based on the axis-aligned cube improves the speed of searching for the local region.

### 3.2. Generate Feature Representation

The feature representation of a point is obtained using an effective encoding strategy that can take advantage of the local structure information. The encoding process for a point *P_o_* is described in detail. The process for other points is similar.

After the local region of the encoded point *P_o_* is determined, all of the points *P_i_* located in the region constitute a point set *S*. It is obvious that the encoded point *P_o_* is located at the center of the local region. All of the points in the set *S* are from the point cloud, and they are called original points.

(2) $$S=\left\{P_{i}|x_{o}-d/2\leq x_{i}\leq x_{o}+d/2,y_{o}-d/2\leq y_{i}\leq y_{o}+d/2,z_{o}-d/2\leq z_{i}\leq z_{o}+d/2\right\}$$

**Figure 3:**
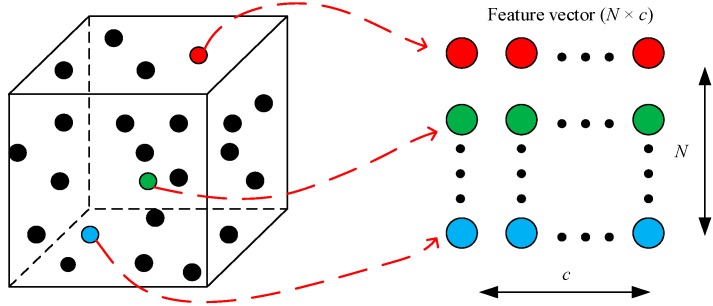
The process of encoding the center point.

Each point in the set *S* has an effect on the center point *P_o_*. To reduce the data size, only *N* points are randomly selected from these points to encode the center point. The center point is encoded into a two-dimensional feature vector. The process of encoding the center point is shown in Figure 3. *c* is the number of the channels used to represent the original point cloud. Each row represents an original point from the local region, and is an element of the feature vector. It is pointed out that the relative positions of the original points in the feature vector are randomly arranged.

For example, four original points are used to construct the feature representation ***F***, and each point is represented using *XYZ* channels. The positions of these four points in the feature representation are random. The ***F*** is shown in mathematical form in (3).

(3) $$F=\left[\begin{array}{ccc} x_{3} & y_{3} & z_{3}\\ x_{2} & y_{2} & z_{2}\\ x_{1} & y_{1} & z_{1}\\ x_{4} & y_{4} & z_{4} \end{array}\right]$$

Because the density of the points may be different across different locations, the point cloud may contain many points in some areas, while it only contains a small number of points in other areas. The number of points included in a local region may be less than *N* if the side length of the cube is too small. In response to this situation, the zero vector with *c*-dimension is added to ensure that the dimension of the feature representation is *N* × *c*.

The virtual zero vector can ensure that there is a big difference between itself and the points in the point cloud. This difference is easy to be recognized by the network and can reduce the impact of the virtual zero vector on the recognition accuracy. In fact, the feature representation is composed of multiple points. A small number of virtual zero vectors have little influence on the recognition accuracy. On the contrary, the virtual zero vectors can increase the diversity of the input data and improve the adaptability of the network to noisy environments. 

### 3.3. Deep Learning Architecture for Processing Feature Representation

**Figure 4:**
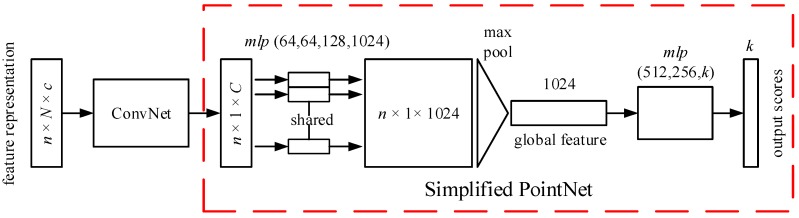
The network architecture for 3D object classification.

**Figure 5:**
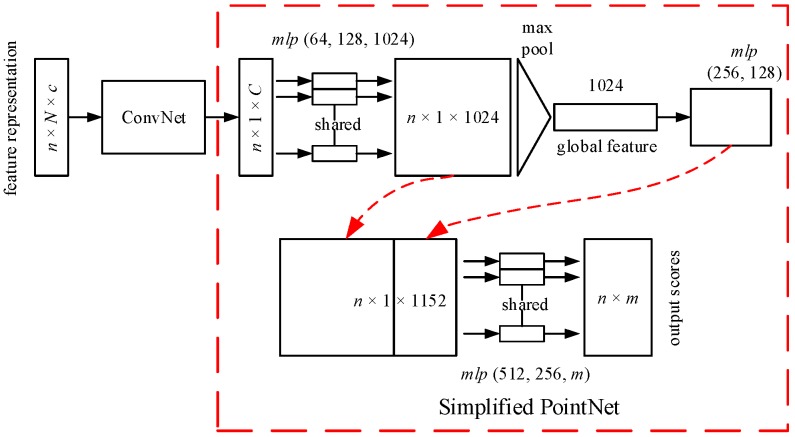
The network architecture for 3D semantic segmentation.

To demonstrate the preponderance of the proposed point cloud encoding method, only a simple deep learning architecture is used in this paper. The deep learning architectures for different tasks are designed based on the PointNet architectures. The structure is marked with a red dotted frame in Figure 4 and Figure 5. The PointNet architectures are different for different tasks. Therefore, the deep learning architectures used in the proposed method are also different for different tasks. For a detailed description of the PointNet, please refer to the literature [11].

The full network architecture for a 3D object classification task is visualized in Figure 4. The input to the network is these feature representations, with dimensions of *n* × *N* × *c*, where *n* is the number of the points entered into the network in one iteration; *N* is the number of the points that are chosen from a local region to construct the feature representation; and *c* indicates the number of the channels of each point. These feature representations are first processed by a simple one-dimensional convolutional neural network block (ConvNet). A new feature vector with dimensions of *n* × 1 × *C* is then obtained. *C* is the channels of the new feature vectors that are input to the simplified PointNet. The shared multilayer perceptron with four layers is then applied to the new feature vector. The max pooling is used to extract the global information. The final classification scores for *k* classes are returned at the end of the network with a fully connected layer.

The full network architecture for a 3D semantic segmentation is given in Figure 5. The architecture is drawn based on the code provided in the literature [11]. It should be noted that a convolutional layer is removed to reduce the model complexity in this paper. These feature representations, with dimensions of *n* × *N* × *c*, are input to the one-dimensional ConvNet. The new features obtained from the ConvNet are entered to the shared multilayer perceptron with three layers. The max pooling is used to obtain a global feature. The global feature is the input of two fully connected layers. The output of the fully connected layer is concatenated with the local feature, with dimensions of *n* × 1 × 1024. Finally, a shared multilayer perceptron with three layers outputs *n* × *m* scores for *n* points and *m* semantic categories.

In these network architectures, *mlp* represents multilayer perceptron networks, and the numbers following it are layer size. The other network settings are exactly the same as those of the PointNet.

The simplified PointNet can be regarded as a set function.

(4) $$f\left(P_{1},\;P_{2},\;\ldots ,\;P_{n}\right)=g\left(\underset{i=1,2,\ldots ,n}{MAX}\left\{h\left(P_{i}\right)\right\}\right)$$

where the *g* and *h* are the *mlp* networks.

The simplified PointNet directly processes the points. For example, given a point *P*_0_ (*x*_0_, *y*_0_, *z*_0_), the *mlp* can be shown in the following style:

(5) $$y=f_{mlp}\left(WP_{0}+b\right)$$

where *f_mlp_* represents the activation function, ***W*** is a weight matrix, and *b* is a bias. The Rectified Linear Unit (Relu) is used as the activation function in the simplified PointNet.

(6) $$f_{mlp}\left(x\right)=\max(0,x)$$

The function *MAX* is a symmetry function to extract the global feature. The function takes ***n*** vectors generated by the *mlp* networks *h*, and returns a new vector ***m*** of the element wise maximum.

(7) $$m=\underset{P_{i}\in S}{MAX}\left\{h\left(P_{i}\right)\right\}$$

## 4. Experimental Studies

The experiments are conducted based on two datasets, namely ModelNet40 and S3DIS. To compare fairly with other methods, the preprocessing of these datasets is consistent with that in Charles et al. [11].

There are 12,311 CAD models in ModelNet40 that contains 40 man-made object categories. These models are split into training sets including 9843 models and test sets including 2468 models. Each point is represented using XYZ channels. Each object contains 1024 points that are uniformly sampled on mesh faces based on face areas. These points are normalized into a unit sphere.

S3DIS contains 3D scans in six areas involving 271 rooms. The complete point cloud is generated from multiple views. Each point has semantic labels from 13 categories including clutter, such as chair, table, and floor. Each point is represented by a 9D vector that includes XYZ, RGB and normalized location as to the room. These points are split by room, which is then sampled into blocks of 1 m × 1 m. In total, 4096 points are randomly sampled in each block during training, and all of the points in each block are used during the test.

The designed network is implemented based on Tensorflow, and the experiments are conducted on Ubuntu 16.04 with a single Nvidia TITAN Xp GPU.

### 4.1. Experimental Setup

To explore the performance of the proposed method on different 3D data analysis tasks, it is respectively applied to 3D object classification and semantic segmentation tasks. The standard PointNet is selected as the baseline. The accuracy is selected as the primary performance metric. The experiments for the classification are completed based on ModelNet40 dataset. The experiments for the semantic segmentation are conducted by using the S3DIS dataset.

To study the effects of these parameters on the proposed method, sensitivity experiments are performed by varying the parameter values. The variation of accuracy with parameters is analyzed in these experiments. The proposed method contains two parameters *d* and *N*. *d* is the side length of the axis-aligned cube. It determines the range of the local region. *N* is the number of points that are used to encode the feature representation. It determines the dimension of the feature representation.

**Figure 6:**
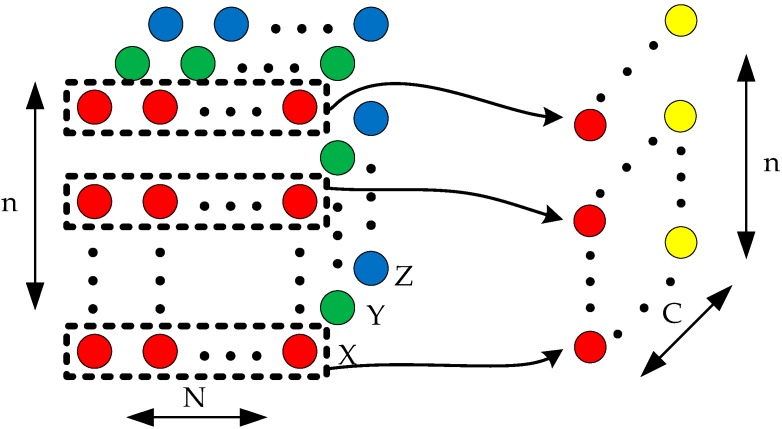
The feature representations are operated by one-dimensional convolution.

In the Tensorflow framework, the operation of the multilayer perceptron is completed by using one-dimensional convolution. Therefore, the data shape is organized according to the requirements of the convolution. For example, the data shape corresponds to the height, width, and channel in the image data. It is assumed that each point is expressed using *XYZ* channels. As shown in Figure 6, the one-dimensional convolution performs a convolution operation on the feature representation by row.

### 4.2. Experiments for 3D Object Classification

In these experiments, the proposed method directly acts on the data provided by Charles et al. [11]. It is fair to compare our experimental results with their results. 

#### 4.2.1. Experimental Results for Classification

In these experiments, the side length of the local axis-aligned cube *d* is set to 0.2. The encoded point is located at the center of the axis-aligned cube. The parameter *N* is set to 2. Two points are randomly selected from all of the points in the local region to encode the center point. If the number of points in the local region is less than two, the zero vector is used to guarantee the consistency of the feature dimensions. Each point is represented using *XYZ* channels for object classification. Therefore, the dimension of the feature representation is 2 × 3 here.

Because only 1024 points are used in each object, the ConvNet takes as input the feature representation with dimensions of 1024 × 2 × 3. The block only contains one convolution layer with 64 output channels. It outputs a new feature with dimensions of 1024 × 1 × 64. The detailed structure for the block is shown in Table 1.

During the training, the batch size is set to 64 to improve the training speed. The maximum epoch is set to 500. The data are augmented on-the-fly by randomly setting the coordinates of some points to 0. To prevent overfitting, the training model is evaluated in each epoch, and only the best training model is saved. Other parameter settings in PointNet are not changed.

The experimental results are shown in Table 2. The overall accuracy (acc_cla) is the classification accuracy for all of the objects. The accuracy avg. class (acc_avg_cla) represents the average class accuracy that is obtained by averaging the accuracy of all categories. Note that all results are presented as a percentage in this paper. The calculation process of overall accuracy and accuracy avg. class is also given in the following equations:

(8) $$acc\_cla=\frac{T}{A}$$

where *T* indicates the number of the objects that are correctly classified, and *A* represents the total number of the objects.

(9) $$acc\_avg\_cla=\frac{1}{M}\sum_{i=1}^{M}\frac{T_{i}}{A_{i}}$$

where *T_i_* indicates the number of the category *i* that are correctly classified, and *A_i_* represents the total number of the category *i*. *M* is the number of the categories.

As shown in Table 2, the proposed method is compared with other traditional methods and deep learning methods. It is obvious that the proposed method obtains a better classification accuracy than traditional methods where a point is not the input format. Moreover, the proposed method performs better than PointNet which uses points as the input. However, the input and feature transformation already exist in the PointNet. PointNet also includes a regularization term in its loss. These additional items make the network very complex. The proposed method without these items is very simple compared with PointNet, but it achieves an enhancement in accuracy. This shows that the proposed point cloud encoding method plays an important role in improving the classification accuracy of the network. The proposed method encodes more local spatial information into the feature representation of each point. These features have stronger ability to represent the point cloud structure of an object. They give the simple networks an opportunity to achieve high accuracy.

#### 4.2.2. Classification Details for Each Category

To show the classification accuracy of each category, the accuracies for individual categories are given in Table 3. The proposed method performs better for some categories such as airplane, bowl, and laptop, while it encounters difficulties in handling some categories such as cup and flowerpot. The quality of the point cloud depends on various factors such as shape, size, and transparency of the objects. The classification performance of the proposed method is related to the specific categories. Therefore, the classification accuracies of the proposed method on different categories are different. However, excluding the flowerpot, the classification accuracies of all categories are greater than or equal to 65%. Among the 40 categories, only 9 categories have classification accuracies below 80%. This shows that the proposed method can effectively identify most object categories.

### 4.3. Experiments for 3D Semantic Segmentation

For a fair comparison, the proposed method is evaluated directly on the S3DIS dataset provided by Charles et al. [11]. Six-fold cross validation is used in the experiments that train six models. For example, to obtain model 1, areas 2−6 are chosen as the training set and area 1 is used as the test set. Six models are evaluated on their corresponding test areas. The overall segmentation accuracy is obtained based on these six models. The overall accuracy (acc_seg) is the point classification accuracy. The intersection over union (IoU) is used to define the average IoU (*mIoU*). *mIoU* is the average point classification accuracy across 13 classes, including clutter.

(10) acc_seg=TC/AC

where *TC* is the number of points that are correctly classified, and *AC* is the total number of points that are predicted.

(11) $$\left\{ \begin{array}{l} IoU_{i}=\frac{TC_{i}}{GT_{i}+AC_{i}-TC_{i}}\\ mIoU=\frac{1}{M}\sum_{i=1}^{M}IoU_{i} \end{array}\right.$$

where *TC_i_* is the number of the points belonging to the category *i* and correctly classified, and *AC_i_* is the total number of the points predicted to belong to the category *i*. *GT_i_* is the ground truth number for the category *i*, and *M* is the total number of the categories. IoU*_i_* is the IoU value for the category *i.*

In these experiments, the side length of the local axis-aligned cube *d* is set to 0.1 m. The parameter *N* is set to 4. Four points in the local region are randomly selected to encode the center point. Each point is represented using *XYZ*, RGB and normalized location as to the room. Therefore, the feature representation dimension of each point is 4 × 9.

Because each room block contains 4096 points, these feature representations with dimensions of 4096 × 4 × 9 are input to the ConvNet. The ConvNet block outputs a new feature with dimensions of 4096 × 1 × 64. The detailed structure for the block is shown in Table 4.

**Figure 7:**
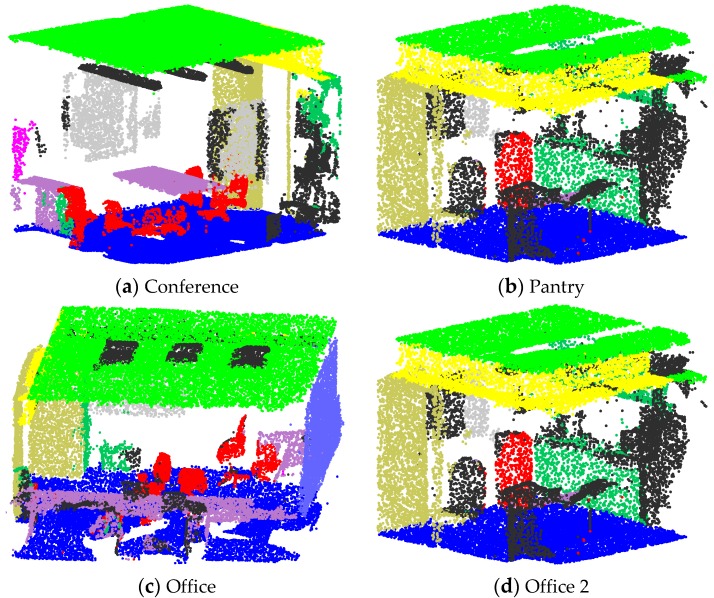
Qualitative results for semantic segmentation.

During the training, the maximum epoch is set to 40. To prevent overfitting, the training model is evaluated for every epoch, and only the best model is saved. Other parameter settings are identical to those in PointNet. The experimental results are shown in Table 5. The semantic segmentation results of some rooms are visualized in Figure 7. Some walls are removed in order to clearly show the indoor objects.

It is clear that the proposed method performs better than PointNet. It is worth noting that the proposed method is combined with a simplified PointNet. The complexity of the simplified PointNet is lower than that of PointNet. However, the proposed method outperforms the PointNet in accuracy and average IoU. It shows that the proposed method effectively utilizes the local structure information of the point cloud. These encoded features provide more discriminative semantic information for the deep learning network. They make it easier for the simple network to recognize the fine-grained features of the objects.

### 4.4. Sensitivity Experiments and Analyses

The proposed method contains two parameters *d* and *N*. The axis-aligned cube side length *d* determines the size of the local region. The feature representation dimension *N* determines the number of the points that are used to build the corresponding feature representation. These experiments are completed for two 3D data analysis tasks based on two different datasets.

#### 4.4.1. Sensitivity Analyses for 3D Object Classification

Sensitivity experiments are conducted based on the ModelNet40 dataset for 3D object classification. The overall accuracy is chosen as the metric in these experiments. Because all of the points are normalized to a unit sphere for ModelNet40, the axis-aligned cube side length is less than one. The parameter *d* is set to 0.1, 0.2, 0.3, and 0.4. The parameter *N* is set to 1, 2, 3, 4, and 5. A total of 20 experiments are conducted in this section.

It should be pointed out that the ConvNet block is slightly different for the feature representations with different dimensions. The specific structure is shown in Table 6. The ConvNet has only one convolution layer when the *N* value is less than three. There are two convolution layers in this block when the *N* value is larger than three. Moreover, only small convolution kernels are used in this block, which guarantees lower network complexity. The moving step size of the convolution kernel is 1 × 1, and the corresponding number of the output channels is 64 for each convolution layer.

**Figure 8:**
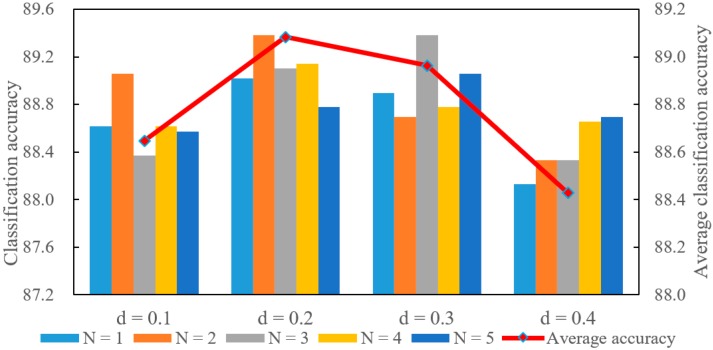
The effects of *d* on classification accuracy.

**Figure 9:**
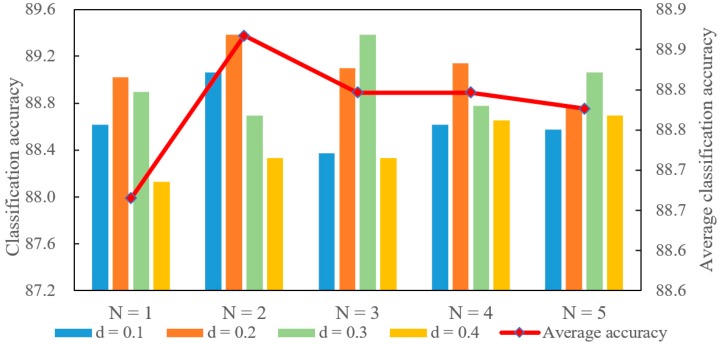
The effects of *N* on classification accuracy.

Each value of the parameter *d* corresponds to multiple values of parameter *N*. Similarly, each *N* also corresponds to different *d* values. Therefore, each parameter corresponds to multiple experimental results. The average for all of these results are used to display the effects of the corresponding parameter. These experimental results are shown in Figure 8and Figure 9. The scale on the left indicates the classification accuracy corresponding to each pair of parameter combinations. The scale on the right indicates the average classification accuracy that is represented using a polyline. The average classification accuracy is selected as the primary analysis result for convenience.

As shown in Figure 8, the average classification accuracy increases when *d* varies from 0.1 to 0.2, and then decreases as *d* varies from 0.2 to 0.4. The local region used to encode a point is very small when *d* is too small. The small local region contains less local spatial information. The local structure information of the point cloud cannot be fully utilized. Therefore, the average classification accuracy is low when *d* is small. However, the accuracy is still low when *d* is very big. The big *d* value means that the local region of the point is big. Therefore, the overlap of the local regions between different points will become large. This makes the feature representations of the different points become similar. The similarity is not conducive to the deep learning network to classify the point cloud objects.

As can be seen from Figure 9, the average classification accuracy increases as *N* varies from 1 to 2 and drops as *N* varies from 2 to 5. *N* is the number of points that are used to encode a point. The feature representation of the point cannot fully take advantage of the local information when *N* is too small. Therefore, the accuracy is low when *N* is small. However, the average classification accuracy drops when more points are used to encode the point. There are two reasons for this situation. On the one hand, more points usually correspond to a large local space. The large local space reduces the difference in feature representations between points. On the other hand, if the local space is small and only contains few points, more zero vectors will be added to the feature representation based on the proposed method. The feature representations become sparse, which is also not beneficial for the deep learning network to classify the objects. Therefore, the average classification accuracy is also low when the *N* value is big.

Comparing Figure 8 and Figure 9, the average classification accuracy shows a similar trend with changes in parameters *d* and *N*. It reflects that there is a strong correlation between these two parameters. The big cube side length *d* builds a large local region. More points are contained in the large local region, which guarantees the density of the feature representation even if *N* is big. However, the large local space and high feature dimension reduce the difference of the feature representations between different points. Similarly, the small local region and low feature dimension cannot make full use of the local spatial information of the point cloud. Therefore, the average classification accuracy drops if these two parameters are too large or too small.

The relationship between these two parameters is further explored based on classification accuracy. If the local region is large but the feature dimension is low, more points are available to construct the feature representation. However, these features utilize a larger space that may reduce the difference between feature representations. Therefore, the classification accuracy is low when the local region is large and the feature dimension is low. In addition, if the local region is small but the feature dimension is high, a small number of points are available and the feature representation may be sparse. This situation is also not conducive to improvement in classification accuracy.

It is clear that these two parameters interact with each other. It is important to ensure that the feature representation is not only dense but also can make proper use of local spatial information. Based on the above analyses, the *d* value is recommended in the interval of [0.2, 0.3] and the *N* value is recommended in the set of {2, 3, 4}.

#### 4.4.2. Sensitivity Analyses for Semantic Segmentation

**Figure 10:**
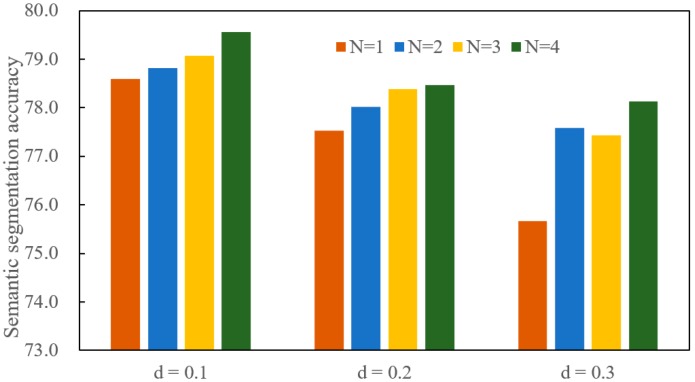
The effects of *d* and *N* on semantic segmentation accuracy.

Several experiments are conducted based on the S3DIS dataset for 3D semantic segmentation. The overall accuracy is selected as the main metric. The rooms are sampled into blocks with 1 m × 1 m for the S3DIS dataset, and the cube side length is less than 1 m. It is noted that semantic segmentation is essentially the classification of the points. It may be disadvantageous to build a large local region. Therefore, the parameter *d* is set to 0.1, 0.2 and 0.3. In addition, the point in S3DIS is represented using a vector with nine dimensions, and the data will take up a lot of computer memory if the *N* is set to very big. The parameter *N* is set to 1, 2, 3, and 4. A total of 12 experiments are completed in this section. These results are not averaged, but are directly shown in Figure 10.

The specific structure of the ConvNet is shown in Table 7. The ConvNet only contains one convolution layer. The convolution kernel size is consistent with the feature dimension, and the size increases as the dimension increases. The convolution stride is 1 × 1, and the corresponding number of the output channels is 64 for each convolution layer.

As shown in Figure 10, semantic segmentation accuracy increases as the *N* varies from 1 to 4 for each fixed *d* value. More points are used to encode the center point when *N* is larger. The feature representation of the center point contains rich local information. The local information is beneficial for the deep learning network to learn the distinguishing semantic information. Therefore, the semantic segmentation accuracy is high when *N* is set a large value.

The semantic segmentation accuracy drops as the *d* value increases when the *N* value is fixed. A Large *d* value means large local region. The feature representation of each point can make use of a large local space. However, the large local space reduces the difference between point features. It is difficult for the deep learning network to distinguish each point when the difference is small. Therefore, the large local region is not conducive to improvement in semantic segmentation accuracy.

The semantic segmentation accuracy obtains the best result when the *d* is set to 0.1 and the *N* is set to 4. It reflects that more points belonging to a small local region can build more distinctive semantic features. The small local space guarantees the spatial difference between point features, and more points ensure the richness of the local information. Therefore, setting smaller local region and bigger feature dimension is beneficial for improving the semantic segmentation accuracy.

### 4.5. Discussions

Local information is critical to improving the performance of deep learning networks. However, the deep learning methods that directly process points cannot effectively use the local information of the point cloud. The proposed method encodes the local information into the new feature representation of each point. Experimental results show that the proposed method obtains high accuracy on 3D classification and semantic segmentation tasks. It is worth noting that the proposed method only uses the simplified PointNet. This suggests that the proposed method can effectively utilize the local information of the point cloud and improve network performance. The proposed method achieves higher accuracy based on ModelNet40 and S3DIS datasets. It suggests that the proposed method has good generalization performance for different datasets on different tasks. The sensitivity experiments suggest that the large local region reduces the difference between point features. The dense point features help improve the accuracy of the deep learning networks.

The axis-aligned cube side length and the feature dimension are two important parameters. They can significantly affect the performance of the proposed method. Moreover, the two parameters interact with each other. It is difficult to choose the optimal combination of these two parameters. Evolutionary algorithms [35] are good at finding the optimal solution of a problem. In the future, the authors will design a suitable objective function to search for the optimal combination of the two parameters.

## 5. Conclusions and Future Work

This paper proposed an effective point cloud encoding method, which enhances the ability of the deep learning network to utilize the local structural information. Two datasets, ModelNet40 and S3DIS, were respectively used for object classification and semantic segmentation. Experimental results shown that the proposed method with the simplified PointNet remained high in accuracy compared with other methods based on complex networks. The main contributions of this paper are summarized as follows:

- The raw point cloud is encoded into the corresponding feature representations with local information. The point cloud encoding method facilitates the deep leaning network to extract more discriminative features.
- The axis-aligned cube that can improve the search speed is used to search for a local region. Partial points in the local region are selected to construct the feature representation of a point with low computation complexity.
- The proposed method reduces the complexity of the network while remaining high in accuracy in classification and segmentation tasks.

The feature dimensions of each point remain the same to meet the requirements of the convolutional networks. The zero vectors are introduced into the feature representation when the number of points in the local region is not enough to construct the feature representation. This may result in sparse features that are not conducive to accuracy improvement. In future work, we will improve the convolutional networks to handle the input data with different dimensions. In addition, the parameters in the proposed method are set manually, and it is also an interesting work to search for the optimal value using evolutionary algorithms.

## Figures and Tables

**Table 1:** The detailed structure of the ConvNet for features with dimensions of 1024 × 2 × 3.

Type	Kernel Size	Stride	Channel
conv	1 × 2	1 × 1	64

**Table 2:** The classification results on ModelNet40.

Method	Input	Views	Accuracy Avg. Class	Overall Accuracy
SPH [19]	mesh	-	68.2	-
3DShapeNets [16]	volume	1	77.3	84.7
VoxNet [9]	volume	12	83.0	85.9
Subvolume [24]	volume	20	86.0	89.2
LFD [16]	image	10	75.5	-
PointNet [11]	point	1	86.2	89.2
Proposed method	point	1	85.5	89.4

**Table 3:** The classification accuracy of each category.

Class	Accuracy	Class	Accuracy	Class	Accuracy
airplane	100.0	dresser	87.2	range_hood	93.0
bathtub	92.0	flower_pot	15.0	sink	65.0
bed	99.0	glass_box	97.0	sofa	97.0
bench	80.0	guitar	96.0	stairs	85.0
bookshelf	97.0	keyboard	95.0	stool	75.0
bottle	96.0	lamp	85.0	table	78.0
bowl	100.0	laptop	100.0	tent	95.0
car	97.0	mantel	94.0	toilet	98.0
chair	96.0	monitor	95.0	tv_stand	88.0
cone	95.0	night_stand	70.9	vase	80.0
cup	65.0	person	80.0	wardrobe	65.0
curtain	80.0	piano	88.0	xbox	85.0
desk	88.4	plant	76.0		
door	85.0	radio	65.0		

**Table 4:** The detailed structure of the ConvNet for feature with dimensions of 4096 × 4 × 9.

Type	Kernel Size	Stride	Channel
conv	1 × 4	1 × 1	64

**Table 5:** The semantic segmentation results on S3DIS dataset.

Method	Average IoU	Overall Accuracy
PointNet [11]	47.71	78.62
Proposed method	**49.04**	**79.57**

**Table 6:** The detailed structure of the ConvNet for different feature dimensions in object classification.

Feature Dimension	Type	Kernel Size	Stride	Channel
1024 × 1 × 3	conv	1 × 1	1 × 1	64
1024 × 2 × 3	conv	1 × 2	1 × 1	64
1024 × 3 × 3	conv	1 × 3	1 × 1	64
1024 × 4 × 3	conv	1 × 3 1 × 2	1 × 1 1 × 1	64 64
1024 × 5 × 3	conv	1 × 3 1 × 3	1 × 1 1 × 1	64 64

**Table 7:** The detailed structure of the ConvNet for different feature dimensions in semantic segmentation.

Feature Dimension	Type	Kernel Size	Stride	Channel
4096 × 1 × 9	conv	1 × 1	1 × 1	64
4096 × 2 × 9	conv	1 × 2	1 × 1	64
4096 × 3 × 9	conv	1 × 3	1 × 1	64
4096 × 4 × 9	conv	1 × 4	1 × 1	64
